# Seasons affect the phosphorylation of pork sarcoplasmic proteins related to meat quality

**DOI:** 10.5713/ab.21.0185

**Published:** 2021-08-25

**Authors:** Xianming Zeng, Xiao Li, Chunbao Li

**Affiliations:** 1 Key Laboratory of Meat Processing and Quality Control, MOE, Nanjing, 210095, China; 2 Key Laboratory of Animal Products Processing, MOA, Nanjing, 210095, China; 3 Jiangsu Synergetic Innovation Center of Meat Production, Processing and Quality Control, Nanjing, 210095, China; 4 College of Food Science and Technology, Nanjing Agricultural University, Nanjing, 210095, China

**Keywords:** Pork, Protein Phosphorylation, Sarcoplasmic Proteins, Season

## Abstract

**Objective:**

Sarcoplasmic proteins include proteins that play critical roles in biological processes of living organisms. How seasons influence biological processes and meat quality of postmortem muscles through the regulation of protein phosphorylation remain to be investigated. In this study, the phosphorylation of sarcoplasmic proteins in pork *longissimus* muscle was investigated in four seasons.

**Methods:**

Sarcoplasmic proteins were extracted from 40 pork carcasses (10 for each season) and analyzed through ProQ Diamond staining for phosphorylation labeling and Sypro Ruby staining for total protein labeling. The pH of muscle, contents of glycogen and ATP were measured at 45 min, 3 h, and 9 h postmortem and the water (P_2b_, P_21_, and P_22_) was measured at 3 h and 9 h.

**Results:**

A total of 21 bands were detected. Band 8 (heat shock cognate 71 kDa protein; heat shock 70 kDa protein 1B) had higher phosphorylation level in summer than that in other seasons at 45 min postmortem. The phosphorylation levels of 3 Bands were significantly different between fast and normal pH decline groups (p<0.05). The phosphorylation levels of 4 bands showed negative associations with immobilized water (P_21_) and positive association with free water (P_22_).

**Conclusion:**

The phosphorylation levels of sarcoplasmic proteins involved in energy metabolism and heat stress response at early postmortem time differed depending on the seasons. These proteins include heat shock protein 70, pyruvate kinase, phosphoglucomutase-1, glucose-6-phosphate isomerase, and carbonic anhydrase 3. High temperatures in summer might result in the phosphorylation of those proteins, leading to pH decline and low water holding capacity.

## INTRODUCTION

The seasons are known to affect meat quality through changes in the energy metabolism, pH decline rate and water-holding capacity [1–4]. High temperatures in summer may induce stress response in pigs through over-expression of chaperones, resulting in the production of poor quality meat [5,6]. Our previous study showed that the incidence of pale, soft, and exudative (PSE) meat is higher in summer than in the other seasons [7].

Glycolytic enzymes affect the rate and extent of glycolysis and postmortem pH decline in porcine muscle [8]. Protein phosphorylation is critical for glycolysis regulation [9], since most of glycolytic enzymes involve phosphate groups. The inactive form of the glycogen phosphorylase kinase, GP b, is transformed into its active form, GP 1, through phosphorylation. High phosphorylation rate of phosphorylase kinase may contribute to fast glycolysis and rapid pH decline in postmortem muscle [10].

Previously, using the same samples as in the present study, we found that the season had significant effects on the phosphorylation of myofibrillar protein, postmortem pH decline, energy metabolism and water mobility [11]. However, little is known about how the phosphorylation of sarcoplasmic proteins in pork is affected by seasons and its association with pH decline and water holding capacity (WHC). The objective of this study was to investigate the phosphorylation of sarcoplasmic proteins of pork *longissimus* muscle in different seasons and relate it to pH decline and water-holding capacity of pork.

## MATERIALS AND METHODS

### Sampling

The porcine muscles used in this study were collected as described previously [12]. Briefly, 40 native black pigs from a commercial farm with the same background were slaughtered in a local slaughterhouse in winter (January, air temperature −2°C to 5°C), spring (March, air temperature 12°C to 15°C), summer (July, air temperature 29°C to 30°C) and autumn (November, air temperature 17°C to 19°C) (n = 10 in each case). *Longissimus lumborum* muscles were obtained and the muscle pH was determined at 45 min, 3 h, and 9 h postmortem. Samples were grouped into normal pH decline group (pH_45 min_>6.0) and fast pH decline group (pH_45 min_<6.0). The 45 min, 3 h, and 9 h muscle samples (10.0 g) were taken and put into liquid nitrogen, then stored at −80°C for proteome analysis.

### pH determination

The pH of the samples was measured by a portable pH meter (Orion Star, Thermo, Waltham, MA, USA), and it was calibrated with two standard buffers of 4.01 and 7.01 before test. In the measurement of each sample, a small hole was made in the sample, and then the electrode was put into the hole until it was immerged in the meat. The pH values were recorded until the reading was stable. Each sample was tested in triplicate.

### Glycogen determination

The glycogen content of the samples was determined by a kit (Jiancheng Bioengineering, Nanjing, China). Three times of volumes of sodium hydroxide (30%, w/v) was added, mixed with 80 to 100 mg of samples in the tube. The mixture was then incubated in hot water (100°C) for 20 min. The mixture was diluted with 16 times of volumes of distilled water after cooling to obtain a 5% (w/v) solution. Subsequently, the mixtures were diluted by mixing 0.1 mL of glycogen solution with 0.9 mL of distilled water and 2 mL of color development reagent. Then, this solution was incubated in the hot water (100°C) for 5 min and cooled to 18°C. Distilled water was used as a control and standard glucose solution was used for quantification. The absorbance at 620 nm was measured (SpectraMax M2e, Molecular Devices, Sunnyvale, CA, USA), The glycogen content was calculated by referring to the standard glucose solution.

### ATP determination

ATP content was determined according to the method of Li et al [11], and each sample was measured in triplicate. Briefly, 5 mL of 7% (v/v) pre-cooling perchloric acid was added into 1.0 g of samples, then the mixture was homogenized for 30 s at 13,500 rpm (Ultra Turrax T25, IKA, Staufen, Germany) and centrifuged for 10 min at 15,000 g and 4°C (Avanti J-E, Beckman, Palo Alto, CA, USA). The supernatant was neutralized with 0.85 M KOH to a pH of 6.8 to 7.0, then centrifuged again for 10 min (15,000 g, 4°C). The supernatant was filtered with a 0.22 μm filter, and 10 μL of filtrate was injected into the HPLC systems (Waters 2965, Waters Technologies, Milford, MA, USA). The C18 chromatograph column (X-Bridge BEH300 C18, Waters Technologies, USA) was applied for separation using 89% elution buffers A (2.5 mM tetrabutylammonium hydrogen sulfate, 0.04 M potassium dihydrogen orthophosphate, and 0.06 M dipotassium hydrogen orthophosphate, pH7.0) and 11% of elution buffer B (methanol). The absorbance was monitored at 254 nm. ATP standards were used for identifying retention time and qualification.

### Water holding capacity

The WHC of the sample was measured according to Li et al [13]. Strips (1.5×1.0×1.0 cm) were cut from the samples along the fiber direction and weighed, which were put into the cylinder glass tube (18 mm in diameter). The tubes were sealed with parafilm membrane before the samples inserted into the probe of the pulsed NMR analyzer (PQ001, Niumag Corporation, Shanghai, China). The resonance frequency of 22.3 MHz, temperature of 32°C was set for the determination. A procedure of CPMG sequence with a τ-value of 200 μs and a total of 3,000 echoes was applied. The scanning repetition was 8 times with an interval of 4,500 ms. The water populations (P_2b_, P_21_, P_22_) were obtained by the program of MultiExp Inv Analysis (Niumag Corporation, China).

### Proteome analysis

#### Protein extraction

Sarcoplasmic proteins were extracted according to Li et al [14]. Briefly, 1.0 g of frozen muscle sample was homogenized in 6.0 mL 100 mM Tris (pH = 8.3). The homogenates were centrifuged, and the supernatant was collected. Protein concentration was determined with the BCA assay (Pierce, Rockford, IL, USA).

#### Sodium dodecyl sulfate-polyacrylamide gel electrophoresis

Five micrograms of samples were loaded onto 4% to 12% Bis-Tris gels (Bio-Rad, Hercules, CA, USA) and electrophorized in 1.0 L of sodium dodecyl sulfate running buffer at 200 V for approximately 1.0 h.

#### Staining and imaging

Gels were stained according to the procedures of Li et al [14]. Briefly, gels were fixed for 12 h in 50% (v/v) ethanol in 10% (v/v) acetic acid. The gels were stained with the Pro-Q Diamond stain for 90 min, and then destained for 30 min in 100 mL of 20% acetonitrile in 0.05 M sodium acetate (pH 4.0). The destained gels were scanned at 532 nm excitation and 580 nm emission (Typhoon TRIO, GE Healthcare, Uppsala, Sweden). After scanning, the gels were stained with SYPRO Ruby for 12 h, followed by destaining in 10% ethanol in 7% acetic acid. The gels were visualized at 532 nm excitation and 610 nm emission and then stained with colloidal Coomassie R-250.

#### Gel image analysis

The band intensities of the gels were quantified using the Quantity One software (Bio-rad, USA). The protein phosphorylation level was calculated dividing the band intensity in the Pro-Q stained image by that in the SYPRO stained image (P/T ratio) [12].

#### In-gel digestion and protein identification

All visible bands were identified using a nano liquid chromatography system (Ultimate 3000, Dionex, Sunnyvale, CA, USA) coupled with the LTQ Orbitrap (Thermo, Bermen, Germany) mass spectrometry. The details were described by Li et al [11].

### Statistical analysis

A mixed analysis of variance model, in which the animal was set as the random effect; and the season and postmortem time were fixed effects, was applied. Means were compared using the Fisher least significant difference method. To evaluate the association between the phosphorylation of sarcoplasmic proteins and meat quality (pH and LF-NMR parameters), the Pearson’s correlation analysis was performed using the program SAS 9.2 (SAS Institute Inc., NC, USA, 2008). The pH, glycogen content, ATP content, and LF-NMR parameters (P_2b_, P_21_, P_22_) were the same as in Li et al [11].

## RESULTS AND DISCUSSION

### pH decline, energy metabolism and water

Both in fast and normal pH decline groups, the pH values, glycogen content and ATP content declined as the time increased (Table 1). The differences in pH values, glycogen content, ATP content and the Water (P_2b_, P_21_, P_22_) between the fast and normal pH decline group at all time points were significant (p<0.05). This indicated that the pH decline rate in 45 min was important in postmortem.

### Phosphorylation of sarcoplasmic proteins

**Figure 1:**
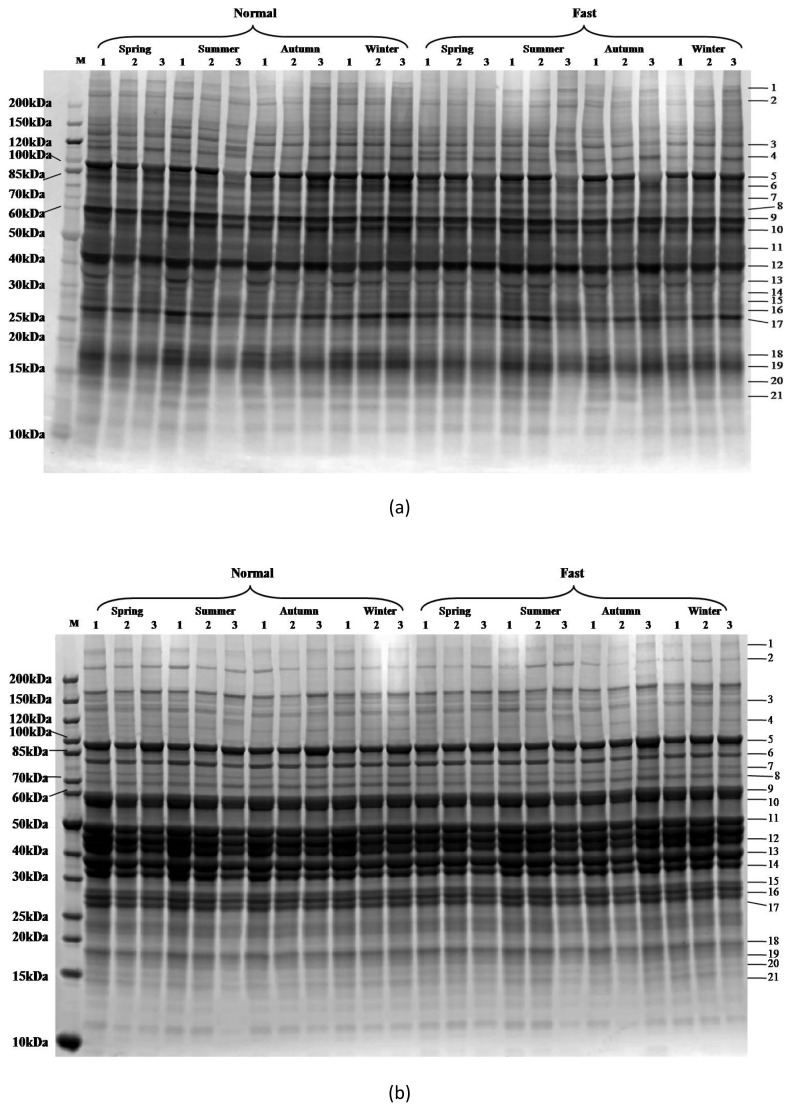
Phosphorylation of sarcoplasmic proteins on SDS-PAGE gels as affected by season and postmortem time. (a) Typical Pro-Q Diamond stained gel. (b) Typical SYPRO Ruby stained gel. 1, 2 and 3 represent postmortem times of 45 min, 3 h, and 9 h. Band numbers were labeled on the right side of the gel. SDS-PAGE, sodium dodecyl sulfate-polyacrylamide gel electrophoresis.

Twenty-one bands were detected (Figure 1). The comparisons between Pro-Q Diamond (Figure 1a) and SYPRO Ruby (Figure 1b) stained images showed differences among samples. The 120 kDa marker band was intensively stained with Pro-Q Diamond and thus, it was used to adjust the variation of different gels. Protein identification results are given in Table 4. The differentially modified proteins were mainly enzymes and chaperones. This indicates that the season might have an influence on the energy metabolism in postmortem muscles through the phosphorylation or dephosphorylation of metabolic enzymes, and further affect meat quality.

**Figure 2:**
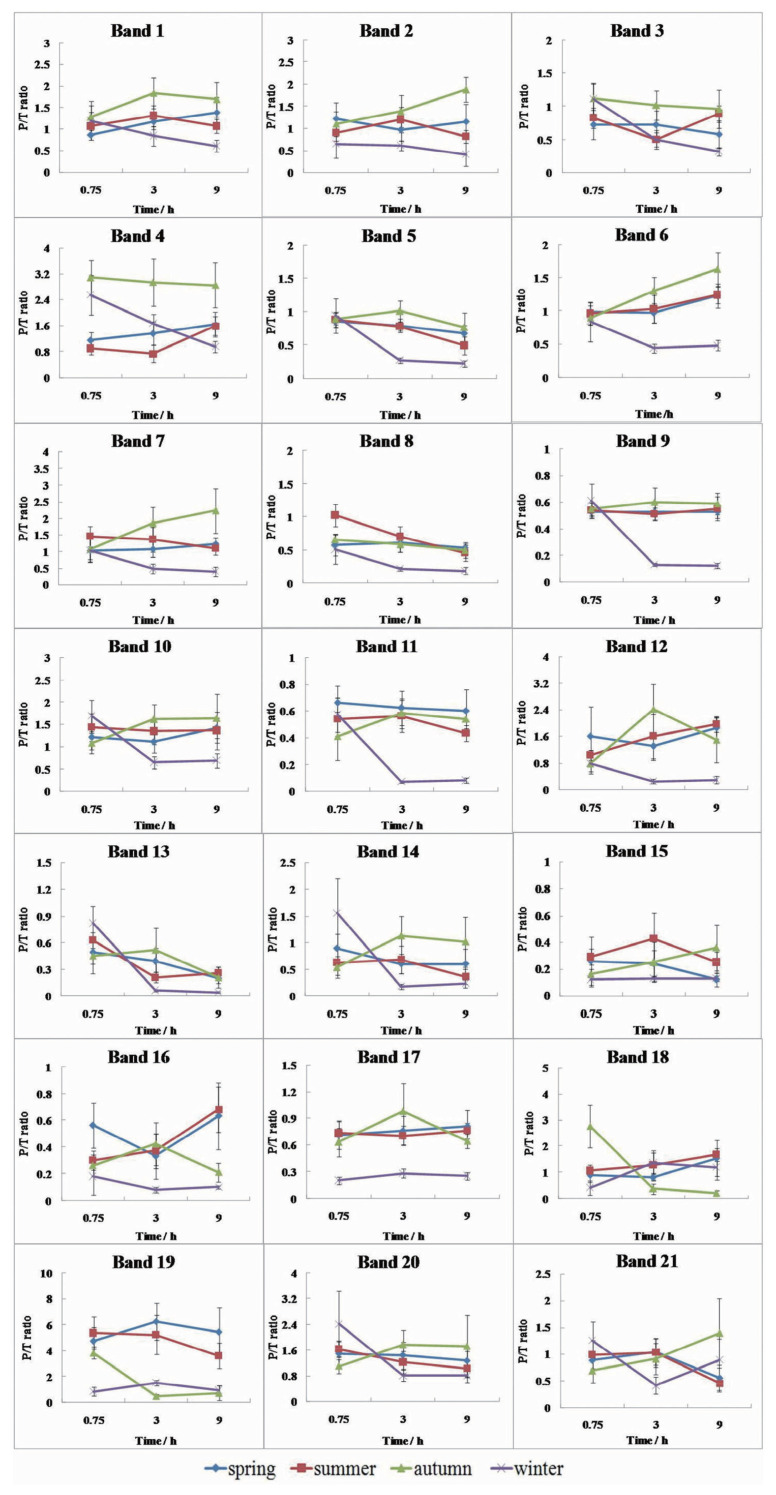
Changes of protein phosphorylation of 21 bands with season and postmortem time. The P/T ratio of these bands was significant influenced by season and/or postmortem time.

The phosphorylation of sarcoplasmic proteins was significantly affected by the season and postmortem time (Table 2, p<0.05). At 45 min, band 4 (sarcoplasmic/endoplasmic reticulum calcium ATPase 1, AT2A1) had higher P/T ratios for the winter and autumn samples than those from spring and summer (Figure 2, p<0.05). Band 8 (heat shock cognate 71 kDa protein, [HSP7C]; heat shock 70 kDa protein 1B [HS71B]) had higher P/T ratio for the summer samples than that of the other groups (p<0.05). HSP7C and HS71B are considered critical proteins for the stress response and regulation of ATP metabolic depletion [15]. HSP70 undergoes phosphorylation under conditions such as exercise and extreme climates to avoid injury [15,16]. In summer, pigs suffer more stress before slaughter, and this leads to faster pH decline and faster energy depletion [11]. At 3 h and 9 h postmortem, the P/T ratios of six of the bands, including bands 5 (glycogen phosphorylase, GP), 6 (ATP-dependent 6-phosphofructokinase, PFKAM), 7 (prolyl endopeptidase, PPCE; heat shock protein HSP 90-alpha, HS90A), 9 (pyruvate kinase, PK; phosphoglucomutase-1, PGM1), 11 (alpha-enolase, ENOA; beta-enolase, ENOB), and 12 (creatine kinase M-type, CK), were much lower for the winter samples. In addition, the P/T ratios of bands 17 (triosephosphate isomerase, TPIS; phosphoglycerate mutase 2, PGAM2) and 19 (heat shock protein beta-1, HSPB1) were also lower for the winter samples than that for the other groups at all-time points. This is because the lower air temperature in the slaughter house may contribute to a faster fall of the carcass temperature during carcass handling before entering the chiller, which would limit enzymes phosphorylation 1.

The differences in air temperature, humidity, transport, and lairage duration may be the main causes for pre-slaughter stress and different meat quality attributes of pork [17,18]. Sommavilla et al [17] observed that the cortisol level is much higher in pigs in summer than that in winter, which is significantly related to higher temperatures and low relative humidity in the summer. Newman et al [18] observed that when pigs are transported in summer and winter with 6-h lairage, the pork pH is high; when the pigs are transported with a 3-h lairage in autumn, the pork pH is high too. However, pH values are within the ones for normal pork. This may be attributed to the relatively better practices in pig slaughtering in those countries.

### Associations between sarcoplasmic protein phosphorylation and pH decline

To explore the association between pH decline and sarcoplasmic protein phosphorylation, the samples were categorized into normal pH decline group (pH_45 min_>6.0) and fast pH decline group (pH_45 min_<6.0). The P/T ratios of bands 8 (HSP7C, HS71B), 9 (PK, PGM1), and 16 (carbonic anhydrase 3, CAH3) were significantly different between fast and normal pH groups (p<0.05, Table 3). These proteins were mainly involved in stress response (HSP7C, HS71B) and energy metabolism (PK, PGM1, CAH3). HSP7C and HS71B are responsible for the stress response and regulation of the ATP supply through protein phosphorylation to avoid injury [15,16]. Pyruvate kinase catalyzes the terminal step of glycolysis to generate ATP [19]. Phosphoglucomutase-1 catalyzes the conversion of glucose-1-phosphate to glucose-6-phosphate and plays a critical role in glycogen metabolism [20]. Carbonic anhydrase catalyzes CO_2_ hydration and promotes the transfer of lactic acid in muscle cells [21]. Thus, these proteins may affect the lactic acid content in muscle and further regulate pH decline.

### Correlation of protein phosphorylation with energy metabolism and water-holding capacity

Correlation analyses indicated that the P/T ratios of seven of the bands were significantly correlated with the pH, nine with glycogen content, and seven with ATP content. The P/T ratios of band 3 (phosphorylase kinase regulatory subunit alpha, KPB1; myosin-binding protein C, MYPC2), band 5 (GP), and band 13 (fructose-bisphosphate aldolase A, ALDOA; glyceraldehyde-3-phosphate dehydrogenase, G3P) were the only ones that positively correlated with pH, ATP, and glycogen contents. The phosphorylation of the three bands presented a decreasing pattern from 45 min to 9 h postmortem (Figure 2). KPB1, MYPC2, GP, ALDOA and G3P had a high coverage in these bands. KPB1 is one of the subunits of the phosphorylase kinase that regulates glycogenolysis in skeletal muscle [22]. GP is the rate-limiting enzymes of the glycolysis, and it can be phosphorylated by phosphorylase kinase on serine 14, which is critical for energy metabolism [10,12]. ALDOA is the enzyme that catalyzes the aldol reactions. The substrate fructose 1,6-bisphosphate is cleaved by aldolase A to produce glyceraldehyde 3-phosphate and dihydroxyacetone phosphate, while dehydrogenase glyceraldehyde phosphatase catalyzes the conversion of glyceraldehyde 3-phosphate to 1,3-bisphosphoglycerate [8]. The highly phosphorylated enzymes at early postmortem may accelerate glycolysis.

The P/T ratio of the 13 bands showed significant correlation with the percentage of bound water in meat (P_2b_, Table 4). Interestingly, only the P/T ratio of band 18 (actin cytoskeletal 3 fragment, ACT3) showed a negative association with P_2b_; ACT3 binds ATP. The P/T ratio of bands 5 and 18 was highly correlated with P_2b_. The P/T ratios of bands 6 (PFKAM), 9 (PK; PGM1; glucose-6-phosphate isomerase, G6PI; heat shock protein 60, HSP60), 12 (CK), and 16 (4-hydroxy-tetrahydrodipicolinate reductase, DAPB; ATP-ADP translocase 1, ADT1; CAH3) showed significant negative associations with the percentages of immobilized water (P_21_) and positive association with that of free water (P_22_). The pH decline ultimately inactivates PFKAM [23], however, this effect may be prevented by phosphorylation [24]. Lametsch et al [25] found that CK could be degraded. Both CK and GP are calpain substrates [26], making calpain a potential enzyme that degrades the two proteins postmortem.

In summary, the seasons have a significant effect on the phosphorylation of heat shock protein 70, pyruvate kinase, phosphoglucomutase-1, glucose-6-phosphate isomerase, and carbonic anhydrase 3 in pig muscles. The P/T ratios of the heat shock protein 70 in summer was much higher than that in other three seasons, which might be due to the high temperature resulting in the phosphorylation of the stress response proteins. This led to faster pH decline and energy depletion, increasing the incidence of PSE meat. Because of the differences in air temperature, relative humidity in four seasons, the phosphorylation of the enzymes involved in energy metabolism (pyruvate kinase, phosphoglucomutase-1, glucose-6-phosphate isomerase, and carbonic anhydrase 3) was influenced. The highly phosphorylated enzymes accelerated glycolysis at early postmortem, led to the lactic accumulation and further pH decline. The P/T ratios of these proteins showed negative association with the WHC of meat, this might be due to the fast pH decline bringing about the low WHC. Thus, measures should be taken to reduce season-induced pre-slaughter stress for obtaining good quality pork.

## Figures and Tables

**Table 1:** Comparison of normal and fast pH decline groups in the pH, glycogen content, ATP content and water (P_2b_, P_21_, P_22_)

Items	45 min		3 h		9 h	
		
	Normal (n = 84)	Fast (n = 36)	Normal (n = 84)	Fast (n = 36)	Normal (n = 84)	Fast (n = 36)
pH	6.31±0.17^a^	5.78±0.10^b^	6.00±0.16^a^	5.61±0.11^b^	5.67±0.10^a^	5.40±0.09^b^
Glycogen (mg/g)	5.88±1.79^a^	4.53±2.2^b^	3.59±1.22^a^	2.35±1.00^b^	1.50±0.88^a^	0.5±0.31^b^
ATP μmol/g	4.89±0.74^a^	3.25±1.55^b^	4.23±1.33^a^	1.34±0.76^b^	1.21±1.01^a^	0.19±0.05^b^
P_2b_ (%)	-	-	3.50±0.34^a^	3.28±0.29^b^	3.31±0.27^a^	3.15±0.30^b^
P_21_ (%)	-	-	96.21±0.57^a^	95.59±1.34^b^	95.61±0.94^a^	94.39±1.43^b^
P_22_ (%)	-	-	0.34±0.34^a^	1.94±1.48^b^	0.99±0.82^a^	2.65±1.28^b^

a,b Different letters indicate significant differences between normal and fast pH decline groups at the same time point.

**Table 2:** Analysis of variance of the phosphorylation levels of bands in response to season and postmortem time

Bands	Season (n = 30 each)				Postmortem time (n = 40 each)			p-value		
		
	Spring	Summer	Autumn	Winter	45 min	3 h	9 h	Season	PT	Interaction
1	1.16±0.31^b^	1.15±0.27^b^	1.60±0.41^a^	0.85±0.36^c^	1.11±0.33	1.29±0.43	1.17±0.49	<0.001	0.256	0.001
2	1.13±0.34^b^	1.00±0.27^b^	1.44±0.51^a^	0.55±0.25^c^	1.01±0.36	1.05±0.38	1.05±0.45	<0.001	0.866	0.016
3	0.67±0.21^bc^	0.74±0.21^b^	1.03±0.25^a^	0.59±0.25^c^	0.94±0.27^a^	0.70±0.27^b^	0.68±0.32^b^	<0.001	0.002	<0.001
4	1.39±0.38^c^	1.01±0.42^d^	2.97±0.63^a^	1.72±0.57^b^	1.91±1.04	1.66±0.89	1.76±0.81	<0.001	0.803	<0.001
5	0.77±0.12^b^	0.72±0.20^b^	0.89±0.19^a^	0.43±0.34^c^	0.89±0.14^a^	0.72±0.29^b^	0.54±0.25^c^	<0.001	<0.001	<0.001
6	1.06±0.18^b^	1.08±0.21^b^	1.27±0.35^a^	0.58±0.25^c^	0.92±0.18^b^	0.95±0.36^b^	1.13±0.44^a^	<0.001	<0.001	<0.001
7	1.12±0.28^b^	1.30±0.33^b^	1.70±0.69^a^	0.63±0.34^c^	1.16±0.37	1.18±0.59	1.24±0.72	<0.001	0.567	<0.001
8	0.58±0.14^b^	0.73±0.28^a^	0.59±0.13^b^	0.30±0.19^c^	0.71±0.26^a^	0.53±0.22^b^	0.42±0.16^c^	<0.001	<0.001	<0.001
9	0.53±0.05^a^	0.54±0.07^a^	0.58±0.08^a^	0.28±0.24^b^	0.56±0.08^a^	0.46±0.19^b^	0.45±0.20^b^	<0.001	<0.001	<0.001
10	1.25±0.27^a^	1.38±0.33^a^	1.45±0.45^a^	0.97±0.52^b^	1.36±0.36	1.17±0.44	1.27±0.49	<0.001	0.337	0.061
11	0.62±0.14^a^	0.51±0.12^b^	0.51±0.23^b^	0.22±0.24^c^	0.54±0.17	0.46±0.19	0.40±0.27	<0.001	0.299	<0.001
12	1.60±0.65^a^	1.59±0.36^a^	1.55±0.88^a^	0.42±0.27^c^	1.10±0.64	1.33±0.45	1.41±0.76	<0.001	0.423	<0.001
13	0.37±0.16^ab^	0.34±0.18^ab^	0.40±0.23^a^	0.23±0.34^b^	0.57±0.21^a^	0.29±0.23^b^	0.18±0.11^c^	0.004	0.002	0.034
14	0.71±0.17^ab^	0.56±0.26^b^	0.89±0.45^a^	0.59±0.15^b^	0.86±0.58	0.64±0.41	0.58±0.42	0.002	0.264	0.008
15	0.21±0.14^b^	0.31±0.13^a^	0.25±0.15^ab^	0.13±0.03^c^	0.21±0.12	0.25±0.16	0.22±0.14	0.043	0.128	0.037
16	0.52±0.28^a^	0.45±0.21^a^	0.30±0.14^b^	0.12±0.08^c^	0.33±0.23	0.29±0.18	0.42±0.30	<0.001	0.115	<0.001
17	0.76±0.16^a^	0.73±0.11^a^	0.76±0.27^a^	0.25±0.05^b^	0.59±0.24	0.69±0.31	0.61±0.25	<0.001	0.819	<0.001
18	1.13±0.57	1.32±0.44	1.21±0.31	1.04±0.05	1.38±1.02^a^	0.99±0.54^b^	1.15±0.72^ab^	0.081	0.043	<0.001
19	5.58±2.01^a^	4.59±1.45^b^	1.84±1.66^c^	1.11±0.44^c^	3.63±2.03	3.34±2.79	2.79±0.62	<0.001	0.096	<0.001
20	1.40±0.38	1.31±0.37	1.52±0.68	1.23±0.88	1.61±0.67^a^	1.30±0.49^b^	1.21±0.62^b^	0.125	<0.001	<0.001
21	0.85±0.28	0.84±0.35	0.99±0.60	0.84±0.46	0.93±0.31	0.86±0.35	0.85±0.16	0.258	0.270	<0.001

a–d Different letters on the same row indicate significant differences between season groups or postmortem time points.

**Table 3:** Analysis of variance of the phosphorylation levels of bands in response to pH decline rate and postmortem time

Bands	Normal (n = 84)	Fast (n = 36)
1	1.20±0.46	1.17±0.35
2	1.05±0.51	1.02±0.37
3	0.79±0.31	0.69±0.31
4	1.86±0.94	1.55±0.82
5	0.71±0.29	0.70±0.20
6	0.97±0.37	1.08±0.28
7	1.16±0.61	1.26±0.47
8	0.51±0.23^b^	0.66±0.25^a^
9	0.46±0.19^b^	0.56±0.09^a^
10	1.27±0.47	1.24±0.34
11	0.46±0.26	0.49±0.18
12	1.18±0.81	1.53±0.71
13	0.36±0.27	0.28±0.17
14	0.74±0.53	0.57±0.34
15	0.21±0.13	0.26±0.16
16	0.30±0.21^b^	0.46±0.27^a^
17	0.61±0.29	0.68±0.19
18	1.16±0.85	1.21±0.66
19	2.87±2.41	4.00±2.13
20	1.35±0.66	1.40±0.52
21	0.91±0.48	0.83±0.33

a,b Different script letters on the same row indicate significant differences between season groups.

**Table 4:** The Pearson correlation analysis between pH, glycogen, ATP, P_2b_, P_21_, P_22_, and the P/T ratio of the 21 bands

Band	pH	Glycogen	ATP	P2b	P21	P22	Protein names
1	−0.11	0.09	0.07	0.23^*^	−0.08	−0.05	Filamin-C
2	−0.08	0.09	0.01	0.30^*^	−0.19	0.003	Myosin-4
3	0.24^*^	0.27^**^	0.22^*^	0.21	−0.27^*^	0.16	Phosphorylase kinase regulatory subunit alpha; Myosin-binding protein C
4	0.06	0.07	0.10	0.22	−0.12	−0.12	Sarcoplasmic/endoplasmic reticulum calcium ATPase1
5	0.30^**^	0.49^***^	0.41^***^	0.42^***^	0.05	−0.12	Glycogen phosphorylase
6	−0.33^***^	−0.13	−0.21^*^	0.21	−0.45^***^	0.32^*^	ATP-dependent 6-phosphofructokinase
7	−0.16	0.09	0.03	0.25^*^	−0.20	0.13	Prolyl endopeptidase; Heat shock protein HSP 90-alpha
8	0.11	0.43^***^	0.25^**^	0.34^**^	−0.001	−0.01	Heat shock cognate 71 kDa protein; Serum albumin; Heat shock 70 kDa protein 1B
9	0.01	0.24^*^	0.10	0.31^**^	−0.34^**^	0.30^*^	Pyruvate kinase; Phosphoglucomutase-1; Glucose-6-phosphate isomerase; Heat shock protein 60
10	0.03	0.11	0.05	0.22	−0.24^*^	0.09	Pyruvate kinase; ATP synthase subunit alpha
11	0.11	0.32^**^	0.15	0.28^*^	−0.14	0.05	Alpha-enolase; Beta-enolase
12	−0.25^*^	−0.10	−0.19	0.24^*^	−0.37^**^	0.34^*^	Creatine kinase M-type
13	0.49^***^	0.58^***^	0.55^***^	0.30^*^	0.03	−0.03	Fructose-bisphosphate aldolase A; Glyceraldehyde-3-phosphate dehydrogenase
14	0.19	0.22^*^	0.25^*^	0.32^*^	−0.05	−0.06	Glycerol-3-phosphate dehydrogenase; L-lactate dehydrogenase A chain; Fructose-bisphosphate aldolase C
15	−0.13	0.10	0.05	0.24	−0.19	0.16	L-lactate dehydrogenase A chain; Glyceraldehyde-3-phosphate dehydrogenase
16	−0.26^**^	−0.04	−0.18	0.06	−0.40^***^	0.53^***^	4-Hydroxy-tetrahydrodipicolinate reductase; ATP-ADP translocase 1; Carbonic anhydrase 3
17	−0.12	0.15	0.05	0.27^*^	−0.20	0.24	Triosephosphate isomerase; Phosphoglycerate mutase 2
18	−0.001	0.001	−0.08	−0.36^**^	0.05	0.11	Actin, cytoskeletal 3 (Fragment)
19	−0.05	0.26^*^	−0.01	0.004	−0.01	0.11	Heat shock protein beta-1
20	−0.20^*^	0.33^***^	0.25^*^	0.27^*^	−0.09	0.14	Myoglobin; Myosin regulatory light chain 2
21	0.15	0.19	0.18	0.20	0.10	−0.12	Profilin-1; Fatty acid-binding protein

Data of 45 min, 3 h and 9 h postmortem were used for correlation with pH, glycogen and ATP, whereas just data of 3 h and 9 h postmortem were used for correlation with P_2b_, P_21_, and P_22_.

*** p<0.001;

** 0.001<p<0.01;

* 0.01<p<0.05.
